# Evaluation of novel starch-deficient mutants of *Chlorella sorokiniana* for hyper-accumulation of lipids

**DOI:** 10.1016/j.algal.2015.08.008

**Published:** 2015-11

**Authors:** Sofie Vonlanthen, David Dauvillée, Saul Purton

**Affiliations:** a Institute of Structural and Molecular Biology, University College London, London WC1E 6BT, UK; b Unité de Glycobiologie Structurale et Fonctionnelle, UMR 8576, CNRS, Université des Sciences et Technologies de Lille, F-59655 Villeneuve d'Ascq, France

**Keywords:** Biofuel, Chlorella, Isoamylase, Microalgae, Starch, Triacylglycerides

## Abstract

When green algae are exposed to physiological stresses such as nutrient deprivation, growth is arrested and the cells channel fixed carbon instead into storage compounds, accumulating first starch granules and then lipid bodies containing triacylglycerides. In recent years there has been significant interest in the commercial exploitation of algal lipids as a sustainable source of biodiesel. Since starch and lipid biosynthesis involves the same C3 precursor pool, it has been proposed that mutations blocking starch accumulation should result in increased lipid yields, and indeed several studies have supported this. The fast-growing, thermotolerant alga *Chlorella sorokiniana* represents an attractive strain for industrial cultivation. We have therefore generated and characterized starch-deficient mutants of *C. sorokiniana* and determined whether lipid levels are increased in these strains under stress conditions. One mutant (ST68) is shown to lack isoamylase, whilst two others (ST3 and ST12) are defective in starch phosphorylase. However, we find no significant change in the accumulation or profile of fatty acids in these mutants compared to the wild-type, suggesting that a failure to accumulate starch per se is not sufficient for the hyper-accumulation of lipid, and that more subtle regulatory steps underlie the partitioning of carbon to the two storage products.

## Highlights

• We have isolated a collection of starch deficient mutants of *Chlorella sorokiniana*.
• We have characterized mutants defective in isoamylase and starch phosphorylase.
• Mutants show no increase in storage lipids as seen for other algal starch mutants.

## 1 Introduction

The unsustainable use of our finite reserves of fossil fuels, and the issues of producing renewable fuels from crop plants given the limitations on available agricultural land, have resulted in major interest in using microalgae as an alternative feedstock for biofuel production [1], [2]. Some microalgal species are particularly attractive as a source of lipid-derived biodiesel given their high growth rates, efficient solar conversion, and tolerance to a wide range of environmental conditions — together with their rich diversity of lipids and ability to accumulate storage lipids to high levels [3], [4]. Accumulation of these neutral lipids occurs under stress conditions such as deprivation of key nutrients (e.g., nitrogen), with the lipids mainly in the form of triacylglycerides (TAGs) that accumulate as lipid bodies within the cell. The extraction and transesterification of the TAGs yield fatty acid methyl esters (FAMEs) that can be used as biodiesel or further processed into bio-jet fuel [5].

In green algae, stress conditions also trigger the accumulation of starch granules in the cells, with starch accumulation preceding the accumulation of lipid bodies following the onset of stress [6], [7]. It is generally assumed that the starch and TAGs serve as electron sinks under conditions where photosynthesis, or metabolism of an exogenous carbon source, is still active but growth is limited [5]. Prolonged stress ultimately results in the breakdown of the photosynthetic membrane and the loss of chlorophyll pigmentation [6]. The maximization of TAG productivity in microalgae therefore requires consideration of both the restricted growth rate under particular stress conditions and the cellular TAG content. Since both starch and TAGs share common precursors in the form of the C3 metabolite pool [8] then it has been proposed that TAG content could be increased by blocking or reducing starch biosynthesis, and thus partitioning carbon towards TAGs.

Several studies have looked at the relationship between TAGs and starch in *Chlamydomonas reinhardtii*; a model alga where starch accumulation has been extensively studied and well-characterized mutants are available [9]. Studies of the sta6 mutant, which accumulates no starch due to a mutation in the small subunit of ADP-glucose pyrophosphorylase (AGPase), have all shown a marked increase in lipid accumulation under nitrogen deprivation when compared to wild type strains [10], [11], [12], [13], [14], [15]. Analysis of other *C. reinhardtii* starch-deficient mutants (i.e., sta1, sta7 and sta11) also indicated a correlation between the amount of starch accumulated under stress conditions and the TAG levels obtained [13], [15]. However, Siaut et al. [7] have questioned these correlations given that they found significant variations in lipid levels among laboratory wild-type strains. They could find no significant difference when comparing sta1, sta6 and sta7 to the presumed parental strain. Nonetheless, studies of starch mutants of other green algal species have also reported hyper-accumulation of lipids when compared to their parental wild-type. de Jaeger et al. [8] found that starchless mutants of the oleaginous species, *Scenedesmus obliquus* showed a clear increase in TAG content compared to the WT without compromising biomass productivity. Similarly, a starchless mutant of *Chlorella pyrenoidosa* showed significant hyper-accumulation of lipid [16], suggesting that the selection for starch mutants of industrially-relevant microalgal species is one strategy towards their “domestication” for mass cultivation [17].

Members of the genus *Chlorella* represent particularly attractive species for such mass cultivation given that they are already cultivated commercially for the health food and cosmetics markets [18], and show key attributes for biodiesel production in terms of robust cultivation in open pond systems and biomass recovery [19], [20]. One species that is particularly suited for industrial cultivation is *Chlorella sorokiniana* [21]. This freshwater species has remarkably short doubling times of only a few hours [22], [23]. It grows optimally at elevated temperatures of 35–40 °C; can tolerate temperatures as high as 46.5 °C and light intensities over 1700 μmol/m^2^/s, and exhibits high biomass productivity [24], [25].

Here we report the isolation and biochemical analysis of starch-deficient mutants of *C. sorokiniana*, including mutants defective in isoamylase and starch phosphorylase. Significantly, we find that these mutants show no increase in TAGs or changes in fatty acid profile, suggesting that the re-engineering of carbon partitioning to favor TAG production is not achieved simply by reducing starch biosynthesis, or that such a strategy is not applicable to all industrial species.

## 2 Materials and methods

### 2.1 Strains and culture conditions

*C. sorokiniana* UTEX1230 was obtained from the University of Texas culture collection. Strains were maintained on tris-acetate-phosphate (TAP) agar plates at 25 °C under constant light [26]. Liquid cultures were grown under constant light (~ 35 μmol/m^2^/s) and agitation (120 rpm) at 25 °C. For induction of starch and triacylglyceride accumulation following nitrogen depletion, the NH_4_Cl in the TAP medium was either reduced to 1/10th of normal (termed TAP-1/10N): final NH_4_Cl concentration of 0.74 mM) or omitted completely (TAP-N).

### 2.2 Isolation of starch mutants

Mutants were isolated following the method described for *C. reinhardtii* [9]. Cells were subject to ultraviolet irradiation to survival rate of 10% and colonies appearing after seven days of growth on solid TAP-1/10N medium were stained directly with iodine vapor. Colonies appearing less stained and not displaying the typical dark blue/purple color, were recovered and restained with iodine to confirm the color change.

### 2.3 Quantification of starch

*C. sorokiniana* was cultivated for five days in 1 L acetate medium with (TAP) or without (TAP-N) nitrogen. The cells were pelleted, washed in water and kept at − 80 °C until use. Cells were lysed by passage twice through a French press at 10,000 psi (with complete breakage confirmed by microscopy), and then centrifuged at 3000 *g* for 20 min at 4 °C. The supernatant was used for measuring total protein using a protein assay kit (Bio-Rad). Starch was extracted from the remaining pellet according to the methods detailed in Delrue et al. [27], using a commercial kit (Enzytec™ kit E1268). Total starch was calculated and expressed as mg starch/mg protein or μg starch/mg cell dry weight. Water soluble polysaccharides (WSP) from the supernatant were also assayed using the Enzytec™ kit.

### 2.4 Sepharose CL-2B gel permeation chromatography

Amylose and amylopectin were separated by gel permeation chromatography on a sepharose CL-2B column equilibrated in 10 mM NaOH as described in Delrue et al. [27]. The optical density of the iodine–polysaccharide complex for each fraction was measured at λ_max_ (maximal absorbance wavelength) after adding iodine solution (1% KI, 0.1% I_2_ w/v) at a dilution of 1:5. The remaining fractions corresponding to the amylopectin were combined and kept at − 20 °C until further analysis of chain length distribution by ion exchange chromatography.

### 2.5 Analysis of water soluble polysaccharides

Water soluble polysaccharides (WSPs) were extracted from the remaining supernatant with chloroform:methanol according to the methods described in Dauvillée et al. [28]. After the removal of the solvent, the dried sample was re-suspended in 10% DMSO (v/v) and loaded on a TSK HW50 gel permeation column, and eluted with 10% DMSO in 500 μL fractions. Each fraction was assayed for total sugars using phenol–sulfuric acid. From each fraction 20 μL was mixed with 20 μL of 5% phenol in a 96-well plate and placed on ice, before addition of 100 μL of concentrated sulfuric acid. The plate was then incubated at 80 °C for 30 min and the absorbance measured at 490 nm. Additionally each fraction was stained by adding iodine solution and the optical density measured as described for fractions separated by CL-2B. Fractions staining red with iodine were combined and kept at − 20 °C until further analysis of chain length distribution.

### 2.6 Chain length distribution

To remove NaOH in amylopectin fractions, as well as DMSO from the WSP fractions recovered from the TSK column, samples were subject to dialysis for 2 h in H_2_O. The solution was then lyophilized and the powder resuspended in 500 μL dH_2_O, 500 μL, and 100 mM sodium acetate (pH 3.5) and incubated at 42 °C. When the sample had reached temperature, 3 μL of isoamylase was added and the reaction incubated at 42 °C overnight. To remove the sodium acetate, samples were passed through carbograph columns (Alltech Deerfield, IL) and eluted with 2 mL 25% (v/v) acetonitrile. The eluted sample was lyophilized and resuspended in 200 μL water before analysis on high performance anion exchange chromatography with pulsed amperometric detection (HPAEC-PAD) (Dionex).

### 2.7 Zymogram analysis of starch enzymes

Crude cell extracts were prepared from a 50 mL mid log phase culture as described in Tunçay et al. [29]. Enzyme activities were monitored through zymogram analysis as detailed in Buléon et al. [30] and Fontaine et al. [31]. Starch synthase was assayed as described in Buléon et al. [30] and Maddelein et al. [32] and phosphoglucomutase activity was monitored as described in Van den Koornhuyse et al. [33]. Phosphoglucose isomerase activity was assayed as described for phosphoglucomutase with the modification of using fructose-6-phophate instead of glucose-1-phophate. Starch modifying activity was assayed according to the methods described in Mouille et al. [38]. For native gels, SDS and β-mercaptoethanol were omitted and gels were electrophoresed at 4 °C. Starch phosphorylase activity was detected on denaturing glycogen containing gels washed 4 times 30 min with 40 mM Tris after the run and one time in 100 mM citrate/NaOH buffer (pH 6.5). They were incubated overnight in the latter in the presence of 20 mM G1P and stained with iodine.

### 2.8 Lipid analysis

Total lipids were measured by direct transesterification to produce fatty acid methyl esters (FAMEs) and analyzed by gas chromatography. Cells were inoculated in 100 mL 1/10N TAP and cultivated for 5 days prior to harvesting. For each sample, 10 mg of dried algae (dried by lyophilization until the measured mass remained constant) was weighed in a 2 mL FastPrep® tube, complete with a ceramic ball and gravel. After addition of 1 mL of MeOH:CHCl_3_:HCl (10:1:1), the tubes were shaken using the FastPrep system at 6 m/s for 30 s prior to incubation at 70 °C for 60 min. The tubes were centrifuged and the supernatant transferred to a 4 mL cryovial. By adding 1 mL of distilled water and 1 mL of CHCl_3_:Hexane (1:4), the phases separated and FAMEs were recovered from the nonpolar upper phase. The samples were analyzed directly on the GC after addition of methyl heptadecanate (C17:0) as an internal standard. The extraction method and identification of FAMEs were developed on a Thermo GC equipped with a Thermo single quadrupole electron impact mass spectrometer (DSQII). A 1 μL sample was injected on a 30 m DB23 column, specifically designed for good separation of FAMEs. The injection temperature was set to 250 °C, at a split of 1:20. The carrier gas was He and used in constant flow of 1.2 mL/min. The temperature of the oven was set at 50 °C for 2 min increased to 180 °C at 15 °C/min, held there for 5 min and then increased to 240 °C at 10 °C/min before a final hold of 2.5 min. The transfer line was set at 250 °C and the MS set to do a full scan of positive ions after 5 min run time between 50 and 750. For quantitation of FAMEs, a standard flame ionization detector (FID) was employed, using the same column, injector and temperature program. The detector was set to 240 °C and nitrogen gas used as make-up gas at 40 mL/min. For thin layer chromatography, total lipid was extracted from 50 mg of lyophilized algal material using chloroform:methanol (2:1 v/v). Layers were separated using water/methanol, the chloroform layer was recovered and samples pipetted onto aluminum backed silica plates. Lipid classes were separated by developing the plate to a solvent front of two thirds in acetone:toluene:water (91:30:3 v/v/v) and fully in hexane:diethyl ether:acetic acid (70:30:1 v/v/v). Lipids were visualized by naphthol staining (0.5% w/v) and sulfuric acid charring.

### 2.9 Electron microscopy

Cells were grown in nitrogen replete or 1/10N TAP medium for five days prior to harvesting 20 mL of culture by centrifugation and resuspended in 0.5 mL of culture medium. Undiluted glutaraldehyde solution (50% in water) and H_2_O_2_ were added to a final concentration of 0.1 vol%. Embedding and preparation for TEM were as described in [34].

## 3 Results

### 3.1 A collection of mutants showing low accumulation of storage starch

**Fig. 1:**
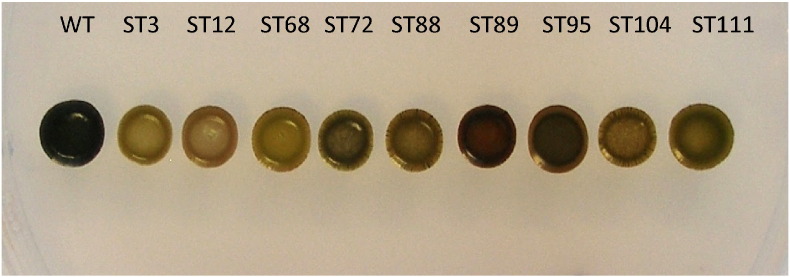
Iodine staining of nine selected mutants isolated after UV irradiation demonstrates a reduction in starch in each strain.

In order to isolate novel starch-deficient mutants of *C. sorokiniana*, we combined UV mutagenesis with a simple iodine-staining method to identify and recover mutagenized colonies that show defective starch accumulation when grown on nitrogen depleted medium [9]. From approximately 2000 screened colonies, 30 potential mutants were recovered and a subset of these was selected for further analysis, following confirmation of their iodine-staining phenotype (Fig. 1).

**Fig. 2:**
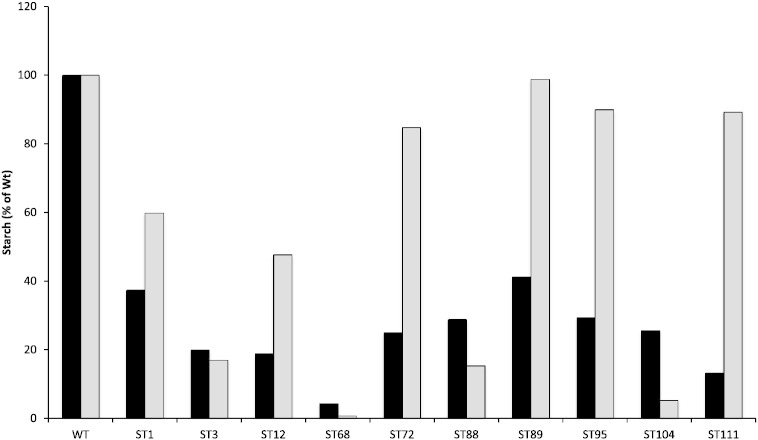
The amount of starch in the mutants relative to the wild-type (WT) is reduced, particularly following nitrogen depletion (black bars). The normalized values for WT (100% ± 4.8%) and ST68 (6.40% ± 0.69%) are from three biological replicates, whilst the other mutants were assayed once. Accurate determination under nitrogen replete conditions (gray bars) is complicated by the low level of starch in the cells.

**Fig. 3:**
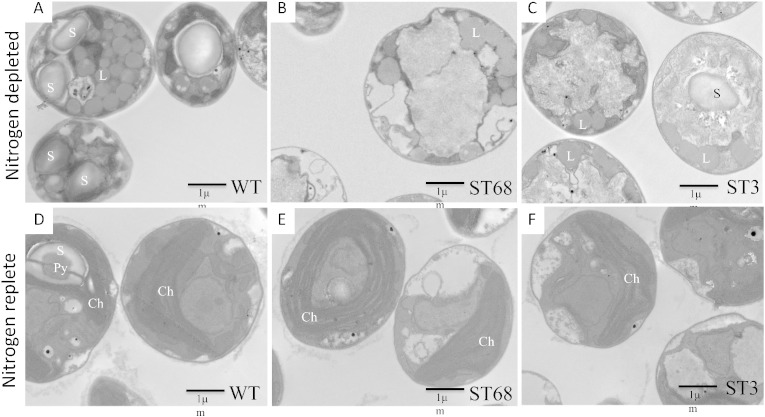
Transmission electron microscopy pictures of cells of WT (A, D), ST68 (B, E) and ST3 (C, F), following cultivation for five days under nitrogen depleted (A–C) or nitrogen replete (D–F) conditions. Under depleted conditions both starch granules (S) and lipid bodies (L) accumulate in the WT. In contrast, only lipid bodies are seen in ST68, whilst in ST3 the amount of starch appears reduced. Under replete conditions, starch around the pyrenoid (Py) of the chloroplast (Ch) is seen only in WT cells.

Direct measurement of starch levels in the wild-type strain and each mutant when grown in nitrogen-depleted medium confirmed that starch accumulation is significantly reduced in all the mutants. In the wild-type, the starch content was determined as 11.5 mg per gram of cell dry weight, whereas starch content in the different mutants was reduced to between approximately 40% and 6% of this value (Fig. 2). Less difference was observed under nitrogen replete conditions, although precise measurements are complicated by the very low amounts of starch under these conditions and high chlorophyll content in the cells (Fig. 2). Mutant ST68 displayed the lowest level of starch under both nitrogen starved and nitrogen replete conditions, with ~ 6% of the wild type level during nitrogen starvation. Transmission electron microscopy of cells from the wild-type and ST68, and also ST3, further supports the starch measurements with lipid accumulation seen in all three lines following nitrogen stress, but no detectable starch granules observed in ST68 and fewer than wild-type in ST3 (Fig. 3 and Supplementary Fig. 1).

### 3.2 ST68 lacks starch debranching activity, and ST3 and ST12 are defective in starch phosphorylase

**Fig. 4:**
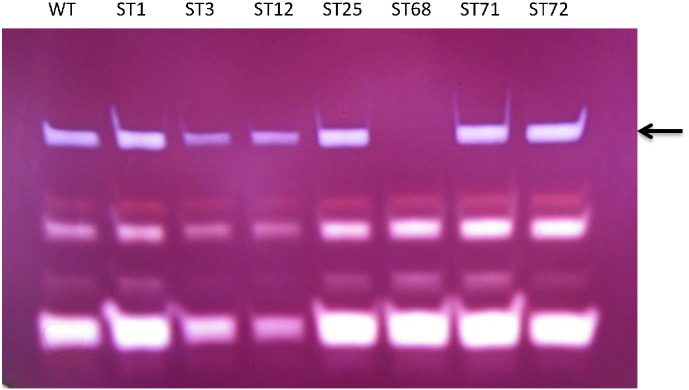
Zymogram detection of starch hydrolytic activities using a polyacrylamide gel containing soluble starch reveals that mutant ST68 lacks isoamylase activity (arrowed).

**Fig. 5:**
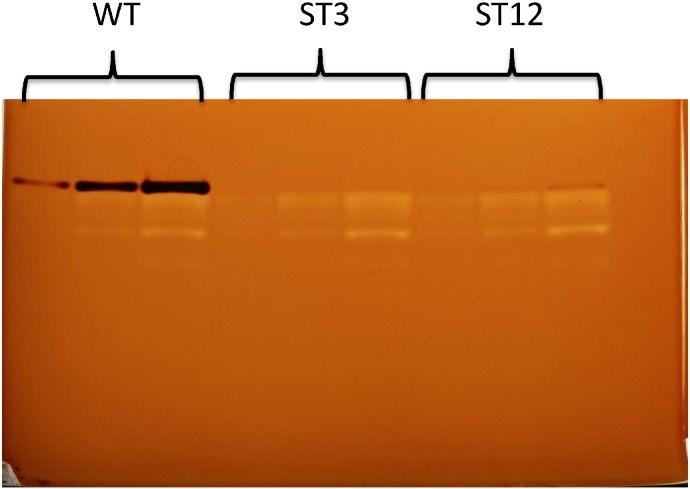
Zymogram analysis of phosphorylase activity in a glycogen containing gel. Three concentrations of protein were used for the wild-type and mutants ST3 and ST12. Phosphorylase activity is greatly reduced in both mutants.

Previous studies of low-starch mutants of *Chlamydomonas* have identified defects in genes for key biosynthesis enzymes including those involved in the formation of the glycan polymers [9] and an isoamylase involved in the debranching of amylopectin — a key step in the formation of the semicrystalline starch granule [35], [36], [37]. The mutants were therefore analyzed using various zymogram-based assays for these enzymes. As shown in Fig. 4, starch-hydrolytic activities are detected following polyacrylamide electrophoresis of cell extracts in gels containing soluble starch. However, a high molecular weight activity is absent from mutant ST68, but is readily detected in the WT and all the other mutants (see also Supplementary Figs. 2 & 3). This activity has previously been shown to correspond to isoamylase and is absent in debranching mutants of *Chlamydomonas* such as sta7 [38]. Additional zymogram analysis for the other starch biosynthesis enzymes: phosphoglucomutase, starch synthases, and starch phosphorylase (see Supplementary Fig. 4), identified two further mutants (ST3 and ST12) that have a significantly reduced activity of starch phosphorylase as shown in Fig. 5. This enzyme catalyzes a reversible reaction in which glucose-1-phosphate (G1P) is used to add a glucose unit to the non-reducing end of an α-1,4-linked glucan chain with the release of inorganic phosphate, or conversely G1P is released from the chain when the enzyme acts in the reverse reaction. Although, plastidial phosphorylases were originally thought to be involved primarily in starch degradation, several mutant studies have indicated a key anabolic role in the formation of starch [39], [40]. A *Chlamydomonas* mutant (sta4) defective in one of two plastidial phosphorylases showed a significant reduction in the amount of storage starch, and with changes to the amylopectin structure and amylose content [39]. The starch structure of ST3 and ST12, together with ST68, was therefore investigated.

### 3.3 ST68, ST3 and ST12 contain a modified starch structure

**Fig. 6:**
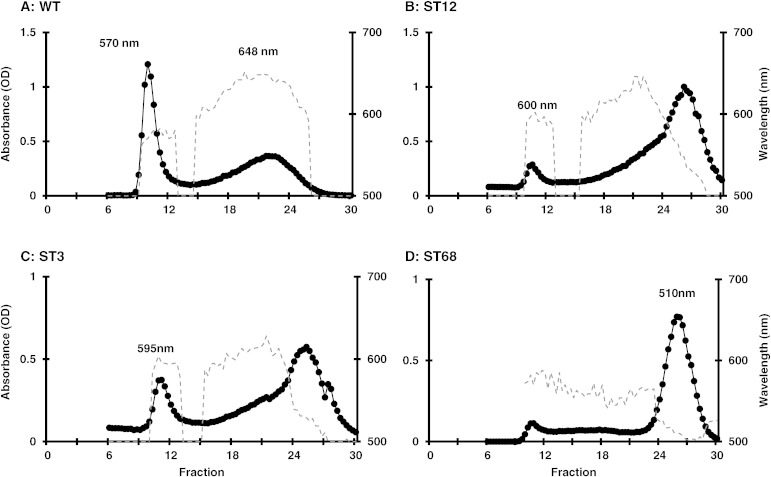
Separation of amylopectin and amylose by sepharose CL2B chromatography. Optical density (black circles) was measured at the λ_max_ wavelength (nm) for that fraction (gray dotted line). Starch from wild-type and mutant strains was extracted from nitrogen deprived cultures. Wild-type profile showing amylopectin with an λ_max_ of 570 nm and amylose at 648 nm. The ST68 mutant displayed a single peak with λ_max_ of 510 nm. Both ST3 and ST12 showed a reduced amount of amylopectin with a higher λ_max_, and replacement of the amylose fraction with a highly heterogeneous fraction containing polymers of different sizes.

The structure and composition of the low amounts of starch present in the three mutants were analyzed by using gel permeation chromatography on sepharose CL2B columns. Iodine staining of eluted fractions from the wild-type strain shows distinct amylopectin and amylose fractions, comparable to that previously found in *Chlamydomonas* [35] with an λ_max_ for the amylopectin and amylose fractions of 570 nm and 648 nm, respectively (Fig. 6A). In contrast, mutant ST68 showed an almost complete absence of the amylopectin fraction, with a new dominant peak eluting late with an λ_max_ of 510 nm, lower than wild-type amylopectin (Fig. 6B). Mutants ST3 and ST12 also display marked reductions in amylopectin, but with an increase in the λ_max_, and the amylose fraction was replaced with a heterogeneous polymer, exhibiting all wavelengths (Fig. 6C and D).

### 3.4 The mutants show accumulation of phytoglycogen

**Fig. 7:**
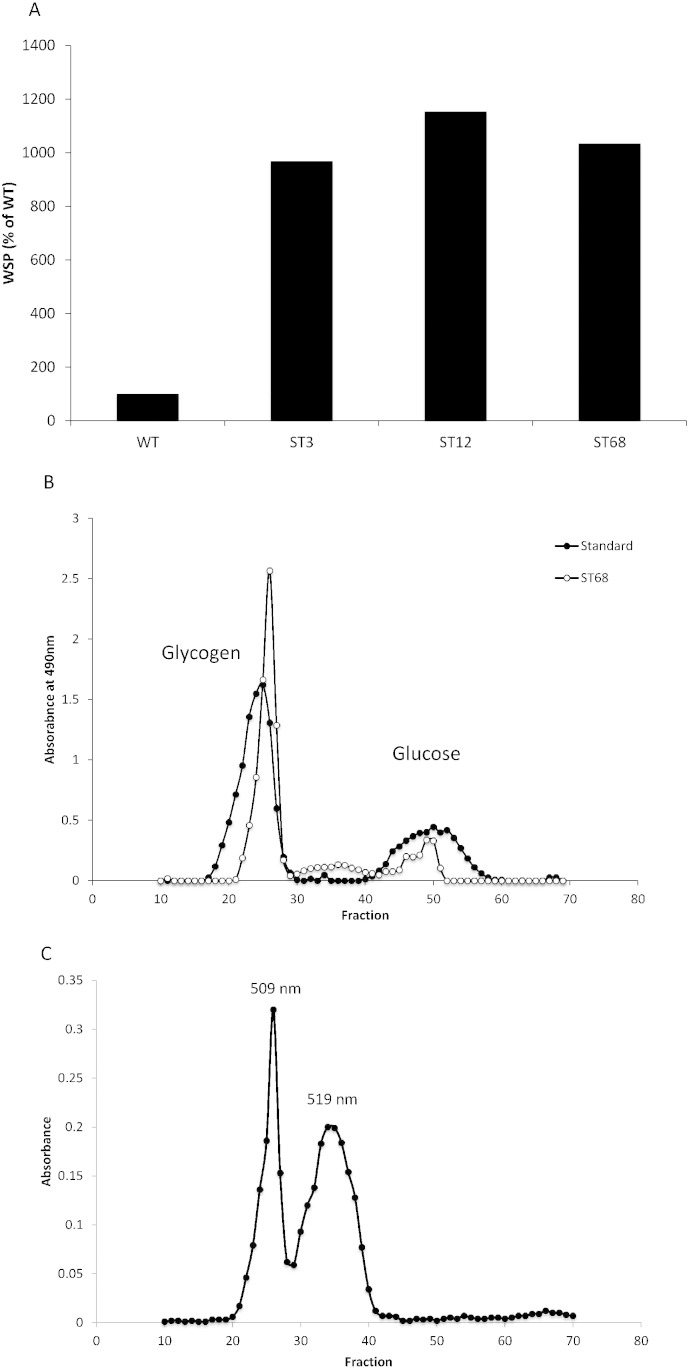
Water soluble polysaccharides (WSP) were extracted from the supernatant of nitrogen deprived cells following lysis and centrifugation to remove insoluble starch. (A). The amount of WSP in each mutant expressed as a percentage of the wild type amount. For ST3 and ST68 the values represent the average of two replicates (914% ± 223% of WT and 745% ± 193% of WT, respectively), with ST12 measured only once. Using the same supernatant from ST68, total sugars were extracted and separated using size exclusion chromatography. Each fraction was then subject to: (B) total sugar analysis by phenol–sulfuric acid staining using a glycogen + glucose standard, with absorbance measured at 490 nm; (C) iodine staining in which the absorbance of each fraction, measured at its λ_max,_ was determined.

All three mutants displayed an order of magnitude increase in water-soluble polysaccharides (WSP) under nitrogen deprived conditions compared to the wild-type strain (Fig. 7A). The WSP from mutant ST68 was extracted with chloroform–methanol and separated by size exclusion chromatography. Fractions collected were subjected to phenol–sulfuric acid determination of total sugars in comparison to a standard of glycogen and glucose (Fig. 7B). The colorimetric determination of total sugars of all fractions revealed a prominent peak eluting between fractions 22–28, similar to glycogen in the standard. A smaller peak was also detected for glucose, however the results indicate that the WSP found in ST68 is more glycogen-like, similar to what has previously been identified in the *Chlamydomonas* debranching mutants sta7 and sta8 [35]. The fractions were also subjected to iodine staining as shown in Fig. 7C. The iodine staining displayed two separate fractions (WSP1 and WSP2); with slightly different λ_max_ of 508 and 519 nm, respectively — both lower than amylopectin (550–570 nm), but not as low as glycogen (490 nm). The two fractions were closely eluting on the column, but the difference in λ_max_ indicates different structures. The two fractions were collected separately (fractions 24–27 for WSP1 and fractions 30–38 for WSP2) and debranched to look at chain length distribution, as described in the next section.

### 3.5 Chain length distribution of the debranched amylopectin and WSP

**Fig. 8:**
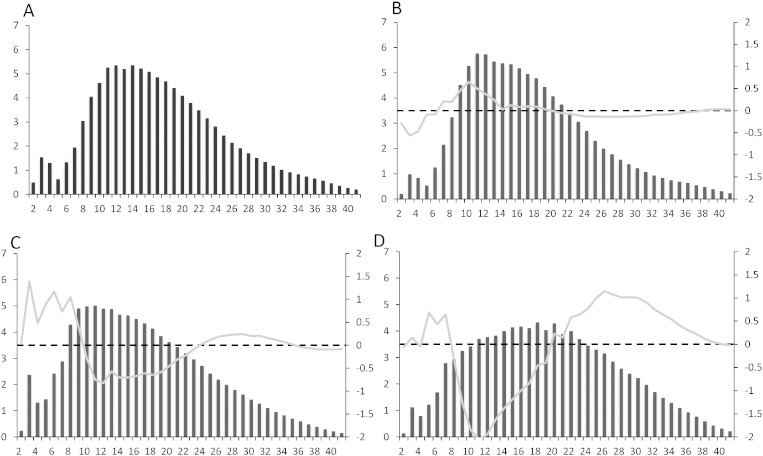
Chain length distribution of wild-type and ST68 mutant amylopectin and water soluble polysaccharides from ST68, after debranching with isoamylase. The results are displayed as percentages of chains of DP 2 to 42. A) WT amylopectin separated by CL-2B. B) Mutant ST68 insoluble starch separated by CL-2B. The black bars represent the relative frequencies of the chains (left y-axis) and the gray line represents the difference in percentage with WT amylopectin (right y-axis). C/D High and low molecular mass WSP from ST68 separated by TSK-HW-50 chromatography. The gray line represents difference compared to insoluble starch isolated from ST68, displayed in (B). Two separate fractions were isolated from TSK-HW-50 and analyzed separately, as WSP1 and WSP2.

After debranching of the amylopectin using isoamylase, chain length distribution (CLD) was analyzed for the WT strain and ST68 by high performance anion exchange chromatography (Fig. 8). The WT strain showed a multimodal distribution, similar to that previously described in *Chlamydomonas* [35], [38]. In the case of ST68, the insoluble low molecular weight polysaccharide eluting late on the CL-2B column was used for analyzing chain length distribution. In contrary to what was expected, the debranched polysaccharide showed a similar chain length distribution to the WT amylopectin (Fig. 8B). This low molecular weight product displayed a dark red color with iodine staining and exhibited a lower λ_max_ than amylopectin indicating a highly branched glucan (as shown in Fig. 6). The late elution on the column however indicates a modified structure with a smaller molecular weight.

The CLD of the WSP extracted from ST68 was analyzed as two separate fractions (WSP1 and WSP2), as eluted from the TSK column. The CLD showed a clear difference between the two fractions, further suggesting that they are composed of differently structured polysaccharides (Fig. 8). The second fraction (WSP2) shows a more even distribution of chain length, whilst WSP1 displays more similarities with the insoluble fraction analyzed from ST68. It is possible that parts of the structurally modified starch in this mutant stay soluble whilst some form insoluble granule-like structures.

### 3.6 The ST68 mutant shows reduced growth in nitrogen deprived conditions

**Fig. 9:**
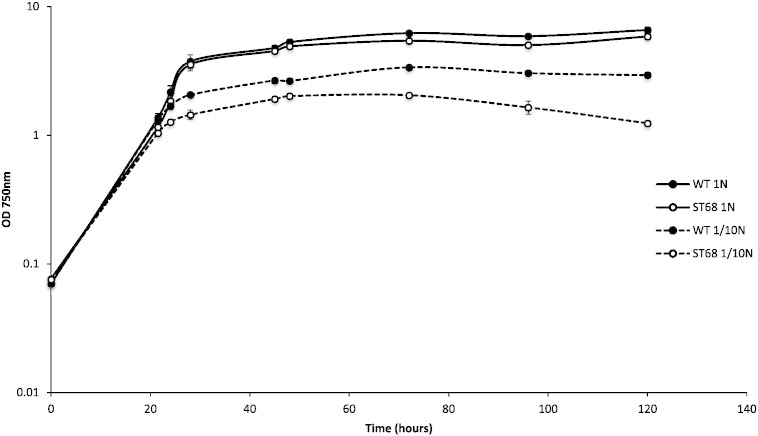
Growth of wild type (WT) (filled circles) and mutant ST68 (empty circles) in standard nitrogen replete medium (1N) (black lines) and nitrogen limiting medium (1/10N) (dashed lines). ST68 shows a more pronounced effect of nitrogen depletion with growth stopping at a lower cell density (OD_750_) and a decline during stationary phase. Error bars represent ± STD (n = 3).

In order to determine whether a defect in starch biosynthesis influences the growth of *C. sorokiniana*, and thus the suitability of such mutants as ‘domesticated’ strains for industrial biotechnology, the growth of ST68 was compared to the WT under both nitrogen replete and nitrogen limiting conditions. No difference in growth performance was observed where nitrogen is in sufficient supply, indicating that the UV mutagenesis has not introduced additional mutations that generally affect the growth rate. However in nitrogen deprived storage starch accumulating conditions, ST68 shows a marked reduction in biomass productivity whereby it enters stationary phase earlier and at a lower cell density when compared to that of WT (Fig. 9). In addition, the chlorosis due to lack of nitrogen was more severe in the mutant (see Supplementary Fig. S7).

### 3.7 Low starch mutants show no hyper-accumulation of lipids or change in fatty acid profile

**Fig. 10:**
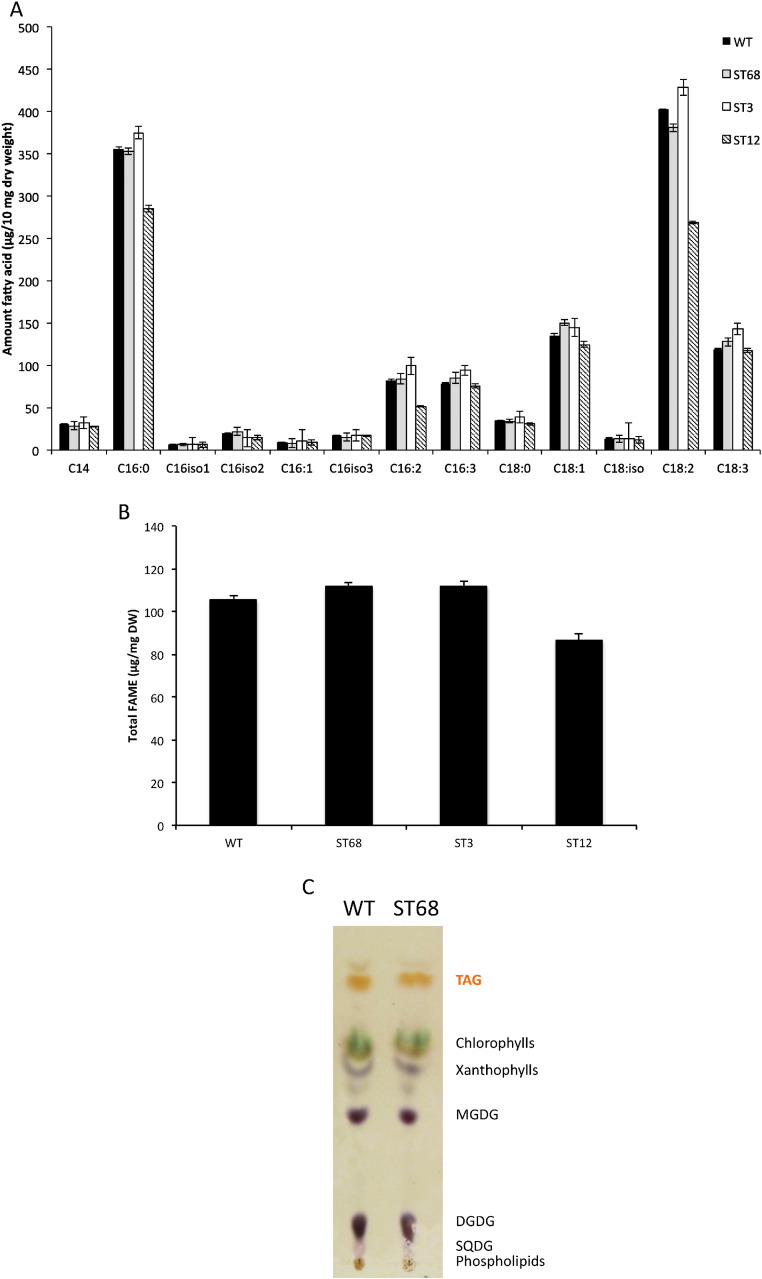
Lipid analysis of wild-type and mutant strains. (A) Total lipid was extracted from 10 mg of dried algae, directly trans-esterified to produce FAMEs and quantified using gas chromatography. Several isomers of C16 could not be confirmed due to lack of comparison standards and data, and were therefore identified only as C16 chains. Error bars represent ± STD (n = 3). (B) The amounts of the individual FAMEs were combined to calculate the total FAMEs per mg of algal dry weight. (C) Thin layer chromatography analysis of total lipids extracted from nitrogen starved cells of WT and mutant ST68. Lipids were separated on silica plates and visualized by naphthol staining and sulfuric acid charring as described in the methods.

As reported for many green algal species [5], *C. sorokiniana* accumulates storage lipids under low nitrogen stress. This is seen in Fig. 3 and in measurements of lipids using either Nile Red staining or thin layer chromatography (Supplementary Figs. S5 and S6). To determine whether there is a hyper-accumulation of lipid in the starch mutants, as described for *Chlamydomonas* [12], [13], [15], total lipids extracted from WT, ST68, ST3 and ST12 were analyzed as fatty acyl methyl esters (FAMEs) by gas chromatography. In contrast to the observations in *Chlamydomonas*, and the recent findings from *Scenedesmus* mutants [8], none of the three *C. sorokiniana* mutants showed any significant increase in FAMEs in comparison to the WT (Fig. 10A). Indeed, ST12 had a lower amount of FAMEs per dry weight compared to the WT. Furthermore, the FA profile in all mutants was not significantly altered, as both WT and mutants accumulated lipids containing mainly C18:2 and C16:0 fatty acids (Fig. 10B). Finally, a direct comparison of triacylglycerides, as opposed to total lipid, by thin layer chromatography confirmed that TAGs accumulate to similar levels in wild-type and mutant strains grown under reduced nitrogen conditions (Fig. 10C).

## 4 Discussion

A key step in the successful exploitation of algal species as a source of lipids, whether as bulk oils for the biofuel sector or specialty oils for the health food sector, is the genetic improvement of what are essentially wild isolates [41]. One obvious strategy for such ‘domestication’ is to increase the carbon flux to storage lipids synthesized under stress conditions by blocking the competing pathway to starch. Several studies of lipid accumulation in starch mutants of *C. reinhardtii* have supported this idea with reported increases in lipid levels as high as ten-fold the wild-type levels [12], [13], [14], [15], [42]. However, other studies have shown that the choice of ‘wild-type’ reference strain can affect the validity of such values given the significant natural variation in accumulated lipid seen between different WT laboratory strains [7]. Indeed, Work et al. [15] found that the isoamylase mutant sta7-10 did show a marked increase in lipid compared to a WT control (strain CC-124), but complementation of the mutant with the wild-type *STA7* gene increased the lipid level further (together with the starch levels), rather than reducing it to the CC-124 level. More recently, Blaby et al. [10] have demonstrated substantial genotypic differences between laboratory strains of *C. reinhardtii.* They further highlighted the complication of the mutant analysis by showing that the presumed parental strain used in several studies of the AGPase mutant sta6 [14], [43] appears to be misidentified, and that the insertional mutation in sta6 also disrupts a neighboring gene involved in metabolism. Nevertheless, their analysis of several independent complemented strains of sta6 does confirm a correlation between hyper-accumulation of TAG in this mutant and the starchless phenotype [10].

Studies of starch mutants in other green algal species also support such a correlation, with reports of increased TAG accumulation in starchless mutants of *S. obliquus* [8] and *C. pyrenoidosa* [16] when compared to the parental strain. This raises the question as to why the *C. sorokiniana* mutants described in this paper show no change in TAG accumulation. The most compelling evidence for increased partitioning of fixed carbon into TAGs has come from the numerous *C. reinhardtii sta6* studies [10], [11], [12], [13], [14], [44] and evidence that the block in starch biosynthesis results in an up-regulation of key enzymes of central carbon metabolism [10]. As shown in Supplementary Fig. S8, the sta6 mutation occurs early in the starch biosynthesis pathway, at the level of AGPase, whereas the ST68, ST3 and ST12 mutations affect enzymes involved in the final stages of the formation of the semi-crystalline starch granule from the polymerized glucan chains. This results in the accumulation of water soluble glucan polymers, but at much lower levels of glucans than that in the starch of the wild-type strain, with ~ 5% being reported in the *C. reinhardtii* isoamylase mutant, sta7 [28], [38]. As such, it cannot be argued that in the ST68, ST3 and ST12 mutants the flux of carbon precursors into glucan polymers remains unchanged and therefore explains the lack of increase in lipid levels. It is possible therefore that the regulatory processes underlying the changes in carbon partitioning in mutants such as sta6 are linked to glucan synthesis rather than simply to an increase in the available pool of carbon precursors. A comparative transcriptomic study of all the available *C. reinhardtii* mutants (Fig. S7) with their complemented equivalents would help to determine if this is the case [10].

Similarly, biochemical analysis of the starch biosynthetic enzymes in the five *Scenedesmus* mutants described by de Jaeger et al. [8], would help our understanding of how to ensure TAG hyper-accumulation without compromising the overall productivity of TAG. Mutant ST68 showed reduced biomass at stationary phase when grown mixotrophically under nitrogen limiting conditions, and thus a lower TAG productivity than the WT — a situation also observed for sta6 [12]. In contrast, the *Scenedesmus* mutants showed no reduction in biomass productivity under phototrophic conditions, resulting in an improvement of TAG productivity of as much as 41% [8].

Clearly, more detailed research into the metabolism of lipids, starch and other hydrocarbons in microalgae is required, and systems biology models of the underlying regulation need to be developed if we are to have the understanding of strain improvement required to make algal biofuels an economic reality.
