# Supplementary material for: Evaluation of novel starch-deficient mutants of Chlorella sorokiniana for hyper-accumulation of lipids
Source: Algal Res. 2015 Nov;12:109–18. doi: 10.1016/j.algal.2015.08.008 (PMC4706179; doi:10.1016/j.algal.2015.08.008)
Supplement: Supplementary file 1 — Supplementary figures. [file mmc1.docx]

**Vonlanthen et al.**

**Supplementary figures**


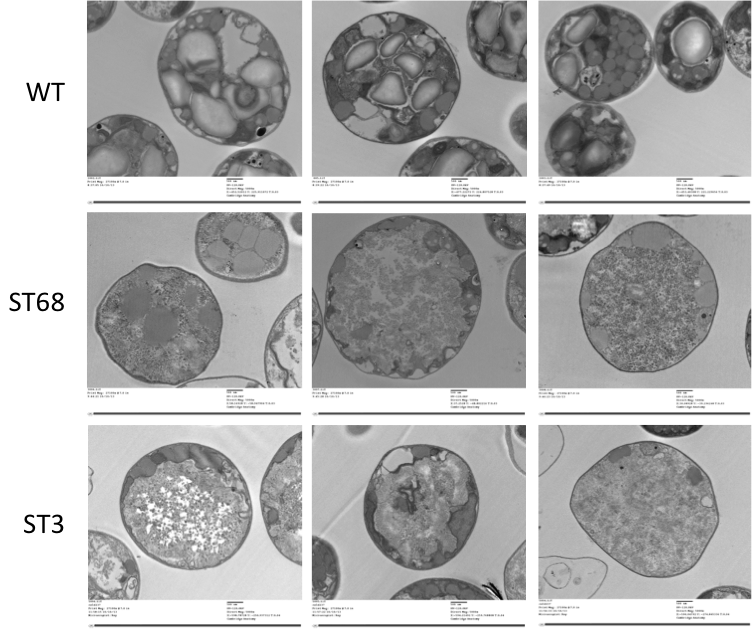


Figure S1. Additional TEM images of WT, ST68 and ST3 cells following cultivation for five days under nitrogen depleted conditions. The images confirm the absence of starch granules in the mutants and the disorganized structure of the cells.

| (A)   |
| --- |
| (B)  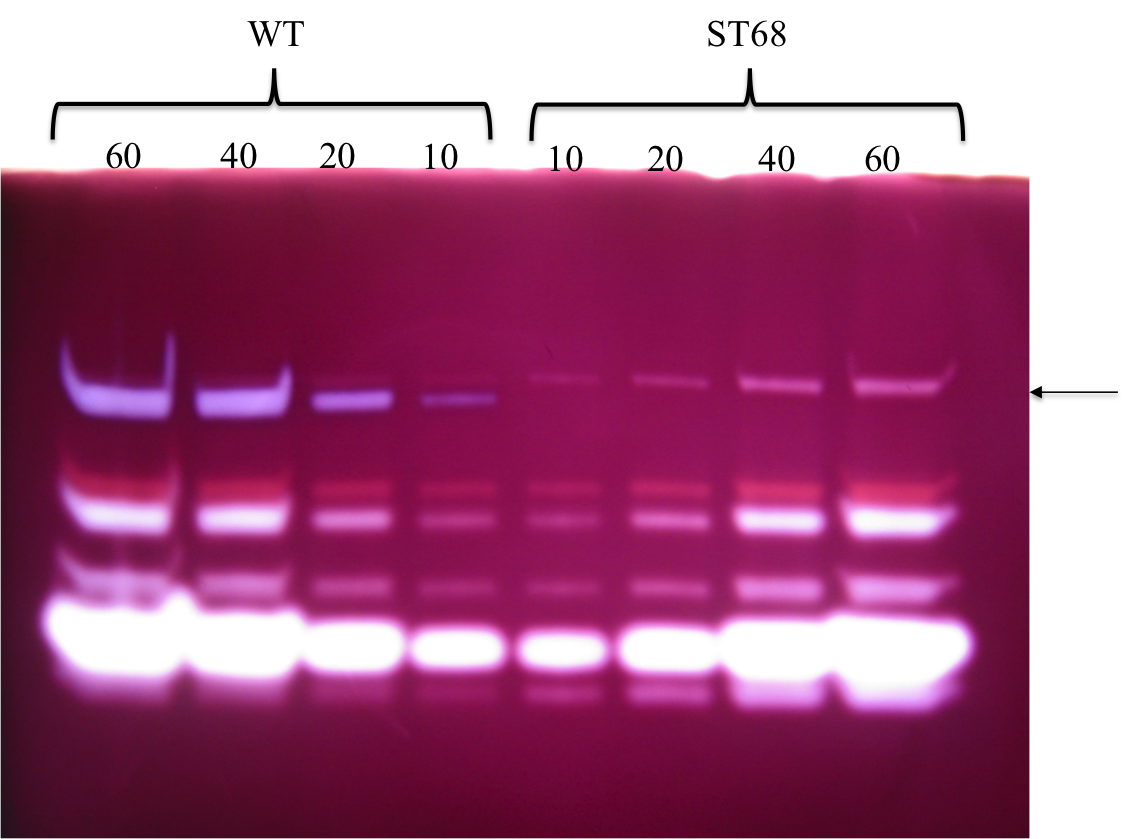 |

| Figure S2. Starch containing zymogram analysis using denaturing gels reveal starch hydrolytic activities and confirms that: (A) the additional mutants have isoamylase activity (highest molecular weight band^[[1]](#footnote-1)^) and give a hydrolytic band pattern comparable to WT. (B) Mutant ST68 completely lacks detectable isoamylase activity (blue upper band in WT) although its absence revealed an underlying minor hydrolytic activity (arrowed). Numbers represent total protein concentrations in µg/mL.  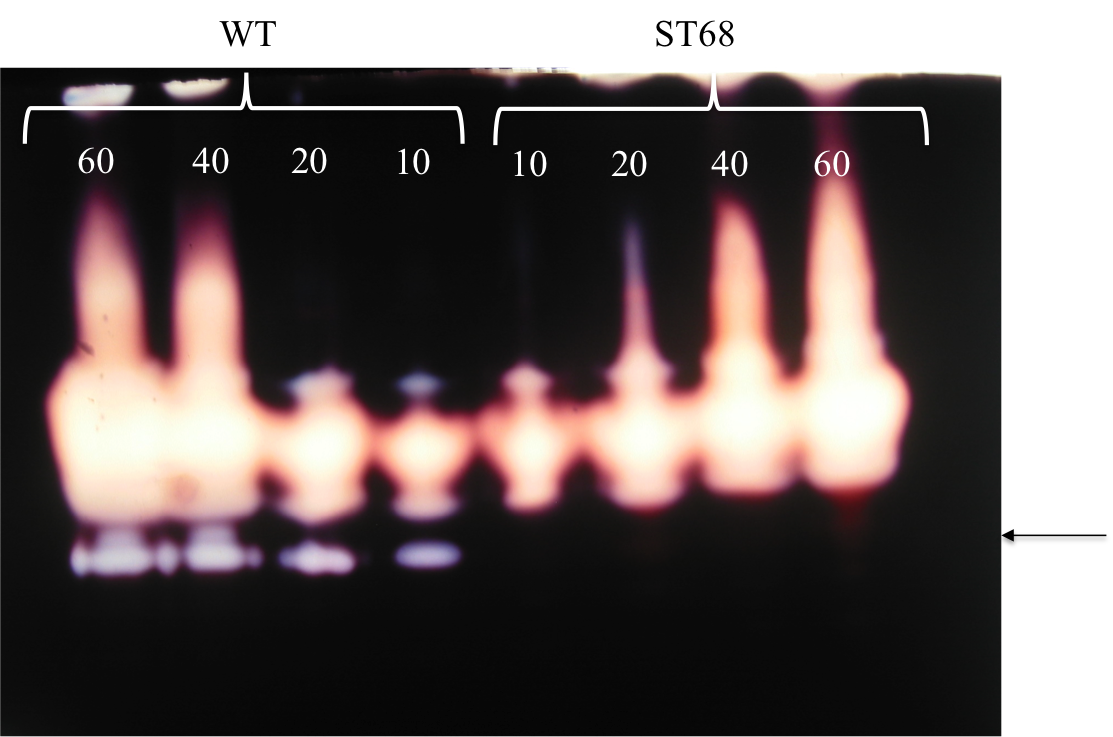  Figure S3. Analysis of ST68 using a non-denaturing starch containing zymogram further supports the absence of debranching activity in ST68 (arrowed). Numbers represent total protein concentrations in µg. |
| --- |

| **Phosphoglucose isomerase and phosphoglucomutase**  |
| --- |
|  |
| **Starch synthase**   |
| **Phosphorylase**   |
|  |
| **D-enzyme**   |

Figure S4. Zymogram analysis of various enzymes in the starch mutants. Phosphoglucose isomerase (A) and phosphoglucomutase (B) activities shown as purple bands on the gel display no differences between mutants and wild type. (C) Starch synthase was assayed by incubating a glycogen-containing gel in buffer containing ADP-glucose, resulting in dark brown bands (indicated by arrows) on a yellow background when stained with iodine as starch synthase enzymes extend the glycogen. Several starch synthase enzymes can be detected in *C. sorokiniana*. (D) Phosphorylase activity assayed in glycogen containing gel. Mutants ST3, ST12, ST88, ST104 and ST111 lack some activity found in WT, as seen by the lack of the dark brown band indicated by the arrow. An activity from WT *Chlamydomonas reinhardtii* is indicated by “+”. (E) Disproportionating-enzyme activity shows reduced activity in ST3 and a lack of activity in ST68 as indicated by the arrow.

Figure S5 (A) Nitrogen starvation in wild-type *C. sorokiniana* results in TAG accumulation. Cultures were grown in TAP medium (nitrogen replete), or nitrogen limited medium (1/10N).

After 48 hours of growth, half of the nitrogen replete cultures were re-suspended in nitrogen free medium. Lipid was measured using Nile Red (NR) fluorescence by adding NR solution to a 3 ml culture sample to give a final concentration of 20 µl/ml. Fluorescence was measured using an LS55 fluorescent spectrometer (Perkin-Elmer) by recording the emission at 586 nm after 2.5 minutes when excited at 510 nm. Error bars represent ± STD (n=3). (B) Comparison of lipid levels between WT and ST68 after 5 days growth in nitrogen limited medium as measured by NR fluorescence as above. Whilst there appears to be a higher lipid level in the mutant, mass spectroscopy and thin layer chromatography (see main text) reveals similar levels of lipids. We therefore conclude that the presence of large starch granules in the WT cells (see figure S1) partially quenches the fluorescence.

Figure S6. **A.** Analysis of wild-type *C. sorokiniana* by thin layer chromatography when grown for 7 days under either nitrogen-replete medium (100% N = 7.4 mM NH_4_^+^) or nitrogen-depleted medium (10% N = 0.74 mM NH_4_^+^), showing the accumulation of TAG and the loss of chlorophyll and the galactolipid MGDG under nitrogen depletion. Total lipids were extracted from 50 mg of freeze dried algae using chloroform:methanol and lipid classes were separated by developing the plate to a solvent front of two thirds in acetone:toluene:water (91:30:3 v/v/v) and fully in hexane:diethyl ether:acetic acid (70:30:1 v/v/v). Lipids were visualized by naphthol staining (0.5% w/v/) and sulfuric acid charring. **B.** Confirmation that the lipid class accumulation in 10%N is TAG by comparison with a C18:1 TAG standard.

|  |
| --- |
| Figure S6. Photograph of cultures after five days of cultivation in nitrogen replete medium (+N) or nitrogen depleted medium with 1/10^th^ the replete level (–N). Although chlorophyll levels in WT and ST68 are the same under N replete conditions, chlorosis due to lack of nitrogen is more severe in ST68 compared to the WT. |

Figure S7. Simplified scheme of biosynthesis of storage lipids and starch. The key starch biosynthesis enzymes are indicated together with known starch mutants of *Chlamydomonas reinhardtii* and the three *Chlorella sorokiniana* mutants described in this work. The *sta6* mutant used for most studies of lipid hyper-accumulation is boxed. The exact role of starch phosphorylase and disproportonating enzyme in starch biosynthesis is not fully understood^[[2]](#footnote-2)^.

1. Mouille et al. (1996) Plant Cell 8: 1353–1366 [↑](#footnote-ref-1)
2. Colleoni et al., (1999) Plant Physiol. 120: 1005-1014 ; Dauvillée et al., (2006) Plant J. 48: 274-285 [↑](#footnote-ref-2)
